# The Ætiology and Rational Treatment of Tetanus; with Cases

**Published:** 1891-07

**Authors:** Geo. H. Lee

**Affiliations:** Galveston


DANIEL'S

### Texas Medical Journal.

A Representative Organ of the Medical Profession, and an Exponent of Rational Medicine; devoted to the Organization, Advancement and Elevation of the Profession in Texas.

PUBLISHED MONTHLY.—SUBSCRIPTION $2.00 A YEAR.

Vol. VII. AUSTIN, JULY, 1891. No. 1.

### Original Articles.

CONTRIBUTED EXCLUSIVELY TO THIS JOURNAL.

*The Articles in this Department are accepted and published with the understanding that we are not responsible for, nor do we indorse the views and opinions of the writers by so doing.*

For Daniel's Texas Medical Journal.

### THE ÆTIOLOGY AND RATIONAL TREATMENT OF TETANUS; WITH CASES.

BY GEO. H. LEE, M. D., GALVESTON.

WHILE in charge of the surgical wards of the John Sealy hospital last year a negro boy, aged 14, with acute traumatic tetanus (evidently from an insignificant wound of the toe, which had cicatrized) came under my care. This boy was treated with the bromides, chloral, morphia, a blister along the spine, and the other usual measures. On the eighth day he became profoundly comatose and died two days later (April 16th, 1890). Two previous cases of traumatic tetanus, which had been treated in this institution since inception (January 10, 1890) had resulted in the same way. While in charge of the negro boy, I became very much interested in the recent literature of this subject, especially in the developments from investigations concerning its bacteriology. Later in the summer I had two other cases in the same hospital, both of which recovered. Full and complete notes of the first were published in the New Orleans *Medical and Surgical Journal* of October, 1890.

I have carefully preserved notes upon my reading, bringing them up to date, together with the second case, hoping to have others, and to be able to add interest to this paper by more extended statistics. I design in this article to place before the profession very briefly and concisely the present status of the investigations concerning tetanus, the resulting conclusions regarding treatment, and the salient points of my two cases.

The bacillus tetani—"a fine linear rod, at one extremity of which is formed a small, colored head, and later a barely colored spore," was demonstrated as existing in the superficial layers of earth, and fatal tetanus was produced by subcutaneous inoculation of mice and guinea pigs (the bacillus being also found at the points of inoculation) by Nicolaier in 1885. Rosenbach found the same bacillus in wounds in man a year later, and produced the disease in mice and guinea pigs by its inoculation. In 1889 Kitasato published the results of certain investigations in the Koch laboratory, in which he succeeded in isolating and in making a culture of this bacillus. Working in the same line a large number of bacteriologists have continued these observations and added to them. As regards the origin of the germ there is much diversity of opinion. Verneuil is the most ardent advocate of the equine-telluric theory in connection with this micro-organism. He holds it "probable that several domestic animals are able to infect man,—that far the most dangerous objects are the horse, with his immediate surroundings, then cultured earth and some of its products." Richelot reports two cases of tetanus after ovariotomy following the spreading of manure in the adjacent hospital court yard. Terillon reports a case developing from a wound of a horse shoe soiled by manure. Bassano in experiments at Marseilles has shown that tetanus can be produced in the lower animals by inoculation of earth from many localities. Th. Kitt concludes an elaborate and careful paper by saying: "From the experiments above described it follows that the traumatic infectious tetanus of the horse is caused by bacilli which resemble the bacilli of the human tetanus, and soil tetanus described by Nacolaier, Kitasato, Buchner and others. Le Blanc, after a series of experiments, decides that "the equine, or bovine origin of tetanus is not proved; if in certain cases its telluric origin is probable, in the large number it is very doubtful." Lefour remarks that in New Hebrides horses are unknown, yet tetanus is very common. M. Trasbot says that at the veterinary school of Alfort 2000 horses are operated on yearly; that he has seen 3400 horses there and only six acquired the disease at the hospital, though there were fifty horses admitted with the disease. No attendant had ever contracted tetanus. These are only a few of the varying opinions of different observers. It may to my mind be accepted as proven that:

a. This bacillus of tetanus does exist in the superficial layers of the earth ; in manure heaps ; and in other decaying vegetable and animal matters.

b. This disease can certainly be produced in the horse, in mice and in other animals by inoculation of such matters.

c. There are quite a number of cases of tetanus in the human being on record, with such a history as would indicate a similar probable origin.

In connection with these conclusions is suggested a very wide and promising field for careful observation and earnest thought, which can only be mentioned. If the specific contagion of tetanus has such an abode and such a distribution, why is this not a very common disease instead of a very rare one? It is necessary simply to refer to the experience of any practitioner of medicine for instances of wounds occurring, continuing and healing under circumstances most favorable for infection without the development of a single tetanic symptom, while in other cases, where the hygiene is the best, where there would seem the least reason to suspect contagion, this disease appears. Thousands of barefoot urchins daily and hourly suffer from wounds upon feet which never are cleansed; wounds which are permitted after to remain filled with earth and refuse matter and heal with no other application; yet no bad symptoms follow, while we find tetanus developing among the best classes, where the hygiene is almost perfect, or in our surgical cases where the strictest asepsis has been observed. How is this explained? We say by the difference in individual susceptibility, by constitutional peculiarities. Just as seed sown broadcast fall upon different soils. Where they find materials favorable for their development they germinate and flourish. Where they fail of these necessary conditions they die. *What are these different subjective conditions in the human system: the individual factors which favor or prevent the development of different diseases?*

In the "Annales de l'Institute Pasteur," early in 1891 appeared an article from the bacteriological laboratory of Val de Grace, by

Drs. Vailards and Vincent, in which they described the organism already made familiar in connection with tetanus, and proceeded to show that the poison which produced the disease was not the bacillus, but a product of the bacillus, a substance neither a ptomain, an alkaloid nor a base, behaving very much like snake poison in that the minutest quantity of the results of certain cultures, when inoculated, gave rise to the disease in its most virulent forms. This substance, they found, was not produced at once in a culture. That until the gelatine commenced to liquify it was incapable of producing toxic symptoms. Inoculations of a susceptible animal with the tetanus bacillus, the toxine being absent and the presence of other germs being guarded against, did not cause tetanus. If though other germs were introduced with the specific micro-organism (freed from toxine), even the ordinary bacillus prodigiosus, or if the wounds after inoculation were exposed to the air so as to become contaminated, toxic symptoms followed. Thus it would seem that the microbe of tetanus is incapable of producing this poisonous substance, the toxine, in a healthy animal, without the presence of another microbe. This is capable of explanation by the protective action of the phagocytes, which, unless interfered with by another bacillus or certain chemical agents, as lactic acid, prevent the tetanus microbe from elaborating its toxine.

Prof. Th. Kitt, of Munich (whose article already referred to was translated and published in the *New Orleans Medical and Surgical Journal*) has shown that these bacilli tetani are not distributed over the body generally, that they exist just about the wound, independent of the presence of pus, and that in this focus the toxine is produced. Yet, after the destruction of the focus the toxine continues in the system and prolongs the tetanic symptoms.

That this disease is infectious in the lower animals has been demonstrated by numerous experiments. Upon its transmissibility from man to man the general opinion is not quite so clear, yet the tendency is decidedly to the affirmative. Paul Berger (*Annual Medical Sciences*, 1889) reports a case observed by Chas. Nelaton and himself, in which there had been a very probable transference of the contagion from a previous case. Dr. Adams relates similar statistics from China, and Richelot observes a similar sequence in two cases. In the same line are recorded many other careful observations which would make it safer to accept the doctrine of the transmissibility of tetanus from man to man as proven.

Very interesting are the results of certain experiments by Drs. Behring and Kitasato upon the production of an immunity to tetanus, which were published in the *Deutsche Medicinische Wochenschrift* (December, 1890), and which are summarized in the *New York Medical Record* (December 6, 1890), and given more fully in the *Medical and Surgical Reporter*, Philadelphia, (January 3, 1891). These experimenters succeeded not only "in conferring an immunity on animals but also in arresting the disease after it had begun to exist." Further they showed that the blood of animals thus rendered immune would by inoculation confer the immunity on other animals, and that it possessed bacteria killing powers for the tetanus germ. Dr. Behring later announced that the substance by which this was accomplished was the trichloride of iodine (*Medical and Surgical Reporter*, January 17, 1891).

### THE RATIONAL TREATMENT.

These developments in the bacteriological field suggest very clearly the indications for the rational treatment of this affection, some of which have given rise to very excellent articles, notably one in the *New Orleans Medical and Surgical Journal* of September, 1890. These indications may be very properly considered under four heads, viz :

1. To remove or destroy the focus where the toxine is elaborated.
2. To relieve the symptoms which result from the presence and continuance of the toxine in the system.
3. To prevent as far as possible all external irritation.
4. To nourish the patient.

In meeting the first indication free incisions about the point of the wound, free curetting if possible, with the use of such germicides as will destroy the bacillus tetani are of first importance; though if the seat of traumatism is such as to admit of conservative amputation this measure is probably more radical. Sormani of Milan experimented to ascertain what agents are most efficient in the destruction of the tetanigenous microbe. He found iodoform, iodol and bi-chloride of mercury absolutely destructive; chloroform, hydrate of chloral and camphorated chloral were extremely efficacious. My preference is for strong solutions of the bi-chloride used for ten to twenty minutes, then carefully removed and a dressing of iodoform freely applied. Sormani (*Lancet*, December 14, 1889), relates a number of cases treated successfully by the local application of iodoform. Another successful case is given in the *University Medical Magazine* (February 1, 1890), by incisions about the wound and iodoform dressing. In the *New Orleans Journal* of June, 1890, Dr. Francisco Pavlini is quoted as recommending injections of a 1 per cent. solution of carbolic acid near the seat of the wound. The author gives the notes of the case of a boy, aged 15, who improved under such injections (quantity not given) every three hours for the first four days, this interval being gradually increased until his recovery. Kitasato (*Annual Medical Sciences*, 1890) found that ten hours immersion in a 5 per cent. solution of carbolic acid did not destroy the virulence of the tetanus germ, while a sublimate solution (one to a thousand) destroyed them in thirty minutes.

2. The relief of the convulsive and tetanic symptons can be best accomplished by remedies which control the reflex centers of the spinal cord (acting as depressants or sedatives of the cord) and which lessen the violent muscular action. If this can be accomplished without interference with cerebration, it will readily be conceded very much the better. Chloral, the bromides, and the preparations of opium are the most popular remedies with the majority of the best of the medical profession. Chloral and the bromides are difficult to administer in such cases, are very mild and slow in action, are apt to be cnmulative and to produce a condition of profound narcosis (as in the case of my negro boy) from which the patient cannot be aroused, and where the second state is worse than the first. The preparations of opium produce congestion of the spinal cord, interfere with digestion, and are open to the objections urged against the preceding remedies. Conium, woorara, cannabis indica, gelsemium and calabar bean have all been used with varying success. The literature of none of them is voluminous or satisfactory. Of calabar bean I will speak especially as the remedy with which I have had some experience. Physostigma is physiologically a powerful depressor of the spinal cord. It of itself does not interfere with cereberation. The nerve ganglia of the heart are stimulated by it, though the heart and respiration are paralyzed by lethal doses. As the sulphate of eserine it can be given hypodermatically, with no danger of irritation from the needle, as often as necessary, in doses accurately adapted to the necessities of the case; and in my hands has been very satisfactory in producing muscular relaxation, a relief from convulsive symptoms, and in promoting rest. To Dr. Thos. Layton, of New Orleans, I owe the suggestion of its use. He reported a successfnl case. Dr. Watson also gives ten successful cases out of eighteen treated with physostigma, presumably in another form. Roemer had twenty recoveries in forty-seven cases. Some observers report serious heart depressions from the use of the extract of physostigma, a complication I have not had.

3. To remove all sources of external irritation, the patient must be placed in a dark room and perfect quiet preserved. De Renzi goes so far as to stuff the patient's ear with cotton, reporting very favorable statistics.

4. In a large proportion of the fatal cases of tetanus the result is due to inanition. To combat this danger it is necessary to give concentrated fluid foods at regular intervals and in sufficient quantities to sustain the vital forces. These should be given by the mouth, when possible, and where this cannot be done, in carefully prepared enemata.

CASE NO. 1.—Traumatic tetanus in a woman of 25 years, due evidently to a suspicious abortion. The treatment consisted in dilating the cervical canal, curetting the uterine cavity with a blunt curette and washing it out with 1 to 2000 bi-chloride solutions. At the time of this operation an offensive discharge was coming from the external os. Little immediate effect from this procedure was noticeable. She was already under the influence of the sulphate of eserine, the administration of which had been commenced two days before with gr. 1-12 hypodermatically. This dose was gradually increased until gr. 1-6 was given. Not much effect was noticed with less than that quantity, and morphia was used in addition ; but under the sixth of a grain hypodermatically very satisfactory rest and relief was produced. No other medications except attention to functions. Isolation, a darkened room, quiet, careful nourishing, were the other features of the treatment. Eserinæ sulphat. gr. 1-6 was given every four hours for twelve days, then the intervals gradually increased. It was the observation of the hospital staff and the physicians who watched this case that just before the time for the injection the tetanic contractions increased very markedly, the teeth being drawn close together; while about 20 minutes after it was given the most thorough muscular relaxation would occur. After four weeks, when the patient was getting only two injections daily, the administration one morning was followed by violent symptoms of collapse, with nausea, vomiting and purging. This occurred on two consecutive occasions, and was attributed to the eserine. The drug was then discontinued. This patient made a satisfactory recovery, but for several months the risus sardonicus was very noticeable.

CASE. No. 2.—Mrs. G. White, aged 25 years, admitted September 12th, a rather thin, medium-sized woman, the mother of several children. History of an abortion a week before, followed by sore throat and a stiffness of the jaws (developed two days before admission). On entering the hospital the jaws were tightly fixed, so were the muscles of the neck and back, while every few minutes violent muscular contractions occurred producing extreme opisthotonos. No tenderness or soreness about the throat. An offensive discharge from the vagina. Temperature 102°; pulse 120. An hypodermic of morphine, gr. ¼ and atrophine 1-150 was given before I saw her. At my visit two hours later eserinæ sulphat. gr. 1-13 was administered subcutaneously, and was repeated every four hours, the dose being gradually increased until the sixteenth, when gr. 1-6 was given. Slight nausea was noted after the first dose. No other bad effects, yet the eserine did not satisfactorily control the painful contractions until 1-6 of a grain was given. On the morning of the 14th chloroform was administered, a hot vaginal douche 1 to 2000 bichloride given, the os widely dilated, the uterine cavity thoroughly curetted (removing some fungosities and some offensive matters), a uterine douche given of hot 1 to 2000 bichloride solution, followed by hot boiled water, and the cavity sponged out with iodine. The patient rested better during the afternoon. The discharge did not reappear. The temperature went down to slightly more than normal. The pulse grew less frequent. The muscular contractions were controlled by the hypodermics of eserine (gr. 1-6 every three to four hours) which were continued until October 5th. The patient was kept in a darkened room. Her diet was milk, milk punches, egg flip, beef tea at intervals of every three to four hours. She had no other medicine except to move the bowels, and a simple cough syrup for an aggravating cough. On October 5th she had improved very much. The interval between injections of eserine was increased, the dose reduced to gr. ⅛. On the 9th she was getting an injection every 12 hours, when it nauseated her very markedly and was discontinued. She was then able to sit in a reclining chair, and made a satisfactory recovery.

In both these cases approximating three hundred hypodermic injections were given without any evidence of irritation from the needle.

